# Ni-Vanuatu health-seeking practices for general health and childhood diarrheal illness: results from a qualitative methods study

**DOI:** 10.1186/s13104-015-1151-7

**Published:** 2015-05-08

**Authors:** Karen File, Mary-Louise McLaws

**Affiliations:** School of Public Health and Community Medicine, UNSW Medicine, UNSW Australia, 3rd Floor Samuels Building, Sydney, NSW 2052 Australia; Epidemiology in Healthcare Infection and Infectious Diseases Control, School of Public Health and Community Medicine, UNSW Medicine, UNSW, Australia, Sydney, Australia

**Keywords:** Childhood, Illness, Diarrhea, Hygiene, Health promotion, Rural, Vanuatu

## Abstract

**Background:**

A local perspective on diarrheal illness has been shown to enhance control strategies for diarrheal disease in traditional rural settings. We aimed to assess caregivers’ understandings of childhood general and diarrheal illness, in one rural community in Vanuatu, to help formulate control strategies for preventing diarrheal disease.

**Findings:**

This was a descriptive study using qualitative analysis of responses to open-ended questions to provide a fuller understanding of illness. Thematic analysis with categories derived from medical anthropology was used to analyse responses and draw conclusions. Twenty-nine participants were interviewed; 22 were maternal responses, three were traditional practitioners, two were rural health care workers, one was a spiritual healer and one had a caregiver role. Respondents categorised illness as biomedical or traditional. Explanations of illness were enmeshed in and derived from both the traditional and biomedical system as the illness experience in the child under their care unfolded. Diarrheal severity influenced treatment selection and respondents expressed a preference for biomedical assistance. Respondents articulated a preference for biomedicine as the primary help-seeking resort for small children. Exclusive reliance on either traditional or biomedical options was uncommon. Local herbal remedies were the preferred home treatment when illness was known or mild, while oral rehydration therapy was used when accessing biomedical practitioners.

**Conclusions:**

Belief about diarrheal illness was influenced by traditional medicine and biomedicine. New evidence points to a growing preference for biomedicine as the first choice for severe childhood diarrheal illness. Diarrheal illness could be countered by maternal hand hygiene education at the medical dispensary and rural aid post.

## Findings

### Background

To prevent diarrhea and reduce mortality in children under two years of age it is critical to obtain early diagnosis and treatment. This has been the focus of social science, as well as applied clinical research for the past 30 years [1]. The psychological processes associated with diarrheal disease in the community are disordered, making the process difficult to understand and translate into health-promotion strategies [2-4]. Improving domestic hygiene practice is potentially one of the most effective means of reducing the global burden of diarrheal diseases in children [5]; however, the cultural contexts in which diarrheal disease occurs are complicated and unique [1,2,6,7]. Commonly, public health promotion focused on approaches that are rational to the biomedical sector but fail to engage traditional communities, because these approaches are often at odds with local traditional beliefs and practices [8]. Health professionals who better understand local interpretations of cause, course and treatment of diarrheal illness are better able to communicate with local people [9-11].

Investigations of respondent perceptions yield local interpretations of illness and help-seeking behaviour and provide an insider’s perspective. Weiss [8] explains “While health professionals concern themselves mainly with dehydration, which they identify as a life-threatening consequence of diarrhea, people in the community may view diarrhea and signs of dehydration as unrelated, as indicators of different diseases with some common features, or they may interpret diarrhea as the consequence of a disease defined by signs and symptoms of dehydration”(p. 8) [8]. Yet, formative research is traditionally undertaken from the biomedical perspective [12]. The researcher transfers their own preconceptions and assumptions which are deeply imbedded in the researchers knowledge systems, not those of the individual or community they are interviewing [2]. This biomedical perspective has created major differences between the therapeutic goals of the practitioner and the person experiencing illness. Local rationalizations may be at odds with empirical evidence of the determinants and prevention of illness. We have used thematic analysis of diarrheal illness to explore for the first time how carers in Epau community seek help for children under five years of age presenting with general and diarrheal illness. This first-hand insight is used to frame appropriate control strategies for preventing diarrheal disease [13-17].

Our research was undertaken in Vanuatu, an archipelago nation in the South Pacific with three official languages: English, French and Bislama (the local creole and lingua franca). “Sixty per cent of all households in Vanuatu speak local languages and 34% speak Bislama as their first language” (p.127) [18]. Distinctly homogenous, being 98% Ni-Vanuatu [18], the Ni-Vanuatu socio-political landscape is hierarchical, organised on the basis of sex and age. British and French colonial rule in Vanuatu in the early 1900s resulted in conversion of the majority of the population to Christianity, the predominant religion. This history has forged links between biomedicine and the church. Although traditional practices, known as *kastom*, were discouraged during colonial times, these have remained strong resulting in the present-day synchronistic system [19,20]. *Kastom*, the local word for traditional knowledge and practice, encompasses a range of social functions from sorcery, reconciliations and wedding gifts [21]. Yet *kastom* has not integrated with biomedicine to the same extent as it has integrated with the church due to associations with black magic that don’t align with Christianity [22].

Like most societies, the health care system in Vanuatu has three social arenas through which illness is experienced; the professional (which we refer to as the biomedical) arena; the folk (referred to as the traditional or *kastom*) arena; and the popular (referred to as home/community) arena [23]. The biomedical arena is the responsibility of Vanuatu Ministry of Health (MoH) under which there are four levels of administration (hospitals, health centers, dispensaries and aid posts) across the country [24]. Dispensaries provide outpatient services in rural areas with a focus on basic essential health care including health promotion and preventive services. Dispensaries are usually staffed by a general nurse who is responsible for supervising aid posts in their area [24]. Within this biomedical arena there are numerous barriers to access and service delivery including cost, distance to services and funding [25,26], while indirect barriers include culture and education. In contrast, Vanuatu’s traditional system is accessible to both rural and urban populations and patient payment is commonly in-kind [22].

In Vanuatu poor hygiene in traditional rural communities results in high morbidity and mortality rates in the paediatric population. The prevalence of diarrheal disease is highest amongst Ni-Vanuatu children aged five and under. The World Health Organisation (WHO) defines diarrhea as three or more loose or watery stools in a 24-hour period [27]. Diarrhea is classified as: acute watery diarrhea that lasts several hours or days; acute bloody diarrhea (dysentery); and persistent diarrhea that lasts 14 days or longer. The symptoms of severe diarrhea include any two of the following signs: lethargic or unconscious; sunken eyes; not able to drink or drinking poorly; and skin pinched goes back very slowly. The Vanuatu Multi Indicator Cluster Survey reported a high prevalence of diarrhea (13.8% of children under five years of age had diarrhea in the two weeks before the survey) with little differences between the urban and rural rates (12.8% vs. 14.1%) (p. 19) [26]. There was no association between diarrhea prevalence, education and the economic status of mothers (p. 19) [26]. About one half (53.7%) of all children with diarrhea accessed Oral Rehydration Therapy (ORT) while the remainder did not access treatment (p. 19) [26].

Our objective was to explore local caregivers’ perspective of childhood diarrheal illness and health seeking beliefs to inform prevention and control strategies of diarrheal disease. The specific objectives were to describe general and diarrheal illness 1) patterns of distress, 2) explanatory models, 3) patterns of help seeking, 4) specific treatments, 5) hand hygiene and 6) make health intervention recommendations.

## Methods

### Study design

This study used a qualitative methodology to conduct individual interviews to document the knowledge attitude and behaviours of caregivers and local health practitioners (traditional and biomedical) toward childhood general and diarrheal illness, and hand hygiene. A pre-determined set of items was used to elicit discussion. The items were framed to include the following concepts around general and diarrheal illness and treatment: *patterns of distress*, *explanatory models*, *patterns of help*-*seeking* and *specific treatments. Patterns of distress* refer to the symptoms people articulate and their experience of the illness [8]. As symptoms may not align with biomedical descriptions of diseases identifying patterns of distress permits comparison of similarities and differences between local and biomedical perspectives. *Explanatory models* capture the meaning of the illness experience. They “specify perceived causes of diarrhea illnesses particular physical, social, supernatural, humoral and other explanations with reference to the underlying system of beliefs in different cultures” (p. 6) [2]. *Patterns of help*-*seeking* are varied spanning biomedical and traditional health systems, religious healers, family or lay community [8]. *Specific treatments* refer to the interpretations of how a particular treatment works with respect to beliefs about the cause of illness [2]. We also explored local hand hygiene knowledge, attitude and practice as a means to reducing diarrheal disease. We then discuss practical strategies for health and hygiene promotion within our findings of local perceptions of diarrheal illness.

### Setting

**Figure 1:**
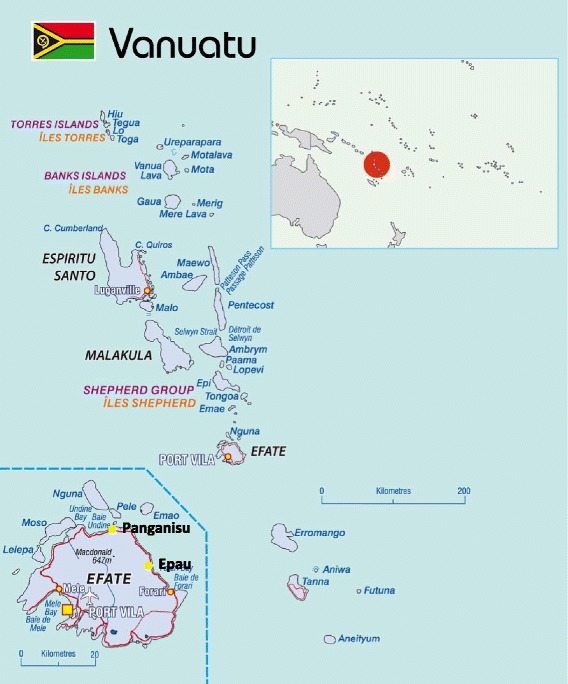
Map of Efate island showing, the location of Epau (Epao) village, the medical dispensary in Panganisu village and Port Vila. (Reproduced with the permission of the Secretariat of the Pacific Community, Noumea, New Caledonia).

Our study site was the rural village of Epau (or Epao) situated in the north of Efate island in Vanuatu (Figure 1). This village is a typical traditional rural coastal village, reliant on subsistence farming. Epau has an aid post where one rural healthcare worker dispenses basic treatments and health advice. Every household has a pit latrine or shares a pit latrine with another household. Epau has a good water supply system in place: one outdoor tap per household. Water tanks store spring and rainwater. There is a river located approximately a ten-minute walk away from the community. Approximately half (52%) of all urban households in Vanuatu dispose of their waste by burning it [18]. With respect to vector control, 88% of all households in Vanuatu have at least one bednet available [18]. Food safety was not observed and data is not available. Epau is situated on a major road with the nearest medical dispensary in Panganisu village 15 minutes away by car. The national hospital with 86 beds, in the capital in Port Vila, is one hour away from Epau by car.

### Participants

Participants purposively selected for interview were individuals who represented several groups who we believed would contribute most to our learning in local interpretations of cause, course and treatment of diarrheal illness. Participants were parents, caregivers and individuals consulted about child illness, such as spiritual leaders, and traditional or biomedical practitioners.

Two groups of individuals were purposively targeted for this study: 22 mothers with children five years and under, one teacher of children under five years old and six healthcare practitioners (two biomedical and three traditional). Twenty-nine participants were interviewed in the following group and individual interviews:

1. one group of 12 mothers with children five and under
2. two groups of mothers with children under five (three mothers in two groups and two mothers in two groups)
3. one group of two rural healthcare workers (one male and one female)
4. three groups of village leaders (two chiefs, one chief’s wife) also *kastom* healers
5. one male teacher also a caregiver
6. one male church leader also a spiritual healer

### Data collection

Interviews were given between August and September 2013, and May 2014, by a trained interviewer with kinship ties to the community. Interviews were conducted in Epau language. Participants provided informed consent, and face-to-face interviews were conducted in homes or yards, at a prearranged time, convenient to each participant. Interviews were continued until no new information emerged. Interviews focused on illness help-seeking behaviour for children five years of age or younger. No material incentives were provided to respondents.

### Data analysis

The local field worker transcribed digital recordings of 29 interviews directly from Epau language to English. Verification of the translations from Epau to English was not undertaken for logistical and financial reasons. Data were coded according to the following anthropological themes used elsewhere [2]: *patterns of distress*, *explanatory models*, *patterns of help*-*seeking* and *specific treatments*. The data were coded using HyperREARCH software [28]. For each theme we report general illness and diarrheal illness. Knowledge, attitude and practice about hand hygiene were also reported.

### Ethical considerations

Ethics approval and community consent were obtained from UNSW Australia Human Research Ethics Committee, the Vanuatu Ministry of Health Ethics Committee, and relevant community leaders and the research participants prior to interviews.

## Results

### Patterns of distress

Participants described their experience of illness as a range of symptoms and distinct diseases. Common symptoms of illness spoken about included “vomiting”, “fever”, “coughing”, ”runny nose” and “headaches”. Biomedical diseases mentioned included “scabies”, “conjunctivitis”, “diarrhea”, “malaria” and “asthma”. Conversely participants also linked visible illness symptoms with imperceptible symptoms or health outcomes. A mother (Participant-13) reported, “Sometimes we cannot tell whether a person is sick until they get so sick and that is when there are signs to show that they are sick… Last year, there was this woman from the community who was taken to the hospital because she did not feel well. On reaching the hospital, she died. She did not look sick to us”. Culture, education and intermittent exposure to biomedicine all make identifying disease challenging.

### Explanatory models

Patterns of distress resulted from not just physical distress but also magic and spiritual matters. Although it was common for participants to articulate a biomedical rationale for good health based on good hygiene and good nutrition, many casual explanations for illness remained vague. One mother (P-14) said, “*I think the children who have scabies are not cared* (for) *properly. They should wash their children properly. Often when parents are busy with work such as gardening*, *they do not give them good care. The children do not get proper nutrition and I think that contributes a lot to the good health of a child*”. Participants’ held a strong belief in *kastom* and illness derived from *kastom*. One mother (P-11) explained how *kastom* illness was real to only local people. “*I will try to explain this one but white people do not believe in it. Whenever a person is talking as if he has gone crazy*, *or if he is lying down and pushing his toes under the sand*, *pillow or mat or in the soil*, *we know that he is being possessed by an evil spirit and I know the kastom leaf that can cure him*”. Bacteria, viruses or life style related diseases were rarely explained.

Diarrhea was described as an illness resulting from “weather” (heat imbalance), “eating too much meat”, “not enough rest”, “black magic” (*kastom* magic) and the natural consequence of milestone (e.g. teething, crawling and walking). For example, one mother (P-8) stated, “*When the weather is really hot and you are sweating and you drink water*, *even if it is clean*, *you can catch diarrhea because you don*‘*t let your body cool off before drinking water*”. Conversely, diarrhea was also said to result from causes apparent to biomedicine such as not washing hands, stress, overwork, flies, contaminated food, foods not prepared properly and bacterial infection. One mother (P-11) explained, “*When I am with my children*, *I make sure that they wash their hands before they eat*”. While another participant (P-14) expressed concern about bacterial infection, “*If an infected child comes to our house*, *we are certainly going to be sick. Everyone in the house is bound to be sick. It is the same as red eye* (conjunctivitis)”.

### Patterns of help-seeking

Participants utilised all three domains of the health care system, seeing help from: 1) local traditional healers (*klevas* and Christian spiritual healers), 2) community and family members (lay people) and 3) biomedical practitioners (rural healthcare workers, nurses and doctors). The importance of seeking help from a biomedical practitioner for severe childhood illness was often expressed. One mother (P-12) explained treatment of mild and severe illness, “*We treat them* (*children*) *with warm water at home and sometimes take them to the aid post. However when it gets severe*, *we take them to the clinic*”. A teacher (P-2) chose biomedicine after the experience of his child dying, “*I am satisfied with their service* (biomedical practitioners). *The treatments they provide work. There are times when I have to charter a transport to take my sick child to be treated in Vila. We learnt a lesson from our child*’*s death*”. Severe sickness was said to arise from delayed help-seeking. The rural health care worker (P-16) stated, “*Few of them go to prayer or use kastom medicine*, *however when the sickness gets severe*, *then that*’*s when they come to me*”. Yet when illness did not improve with biomedical treatment participants sought traditional help. As this mother (P-11) stated, “*When a person is sick and taken to the hospital and yet he does not get better*, *I know that it needs traditional methods to treat that sickness*”.

Health practitioners from all three arenas treat diarrheal illness. Lay people and traditional practitioners with home remedies or *kastom* medicine commonly treat minor diarrheal illness. Biomedical practitioners almost always treat severe childhood diarrheal illness. As one chief (P-21) reiterated, “*There was diarrhea going around that time. We thought that treating her at home was going to work out. We even gave her coconut juice but it did not help*, *she was just seven months old after all. I believe that if she was taken to the health centre earlier*, *it would not have gone that bad*, *also if she was breastfeeding*; *it would have helped a lot*”. Participants expressed a belief that the biomedical practitioner successfully heals babies.

### Specific treatments

Treatments fall into the following broad biomedical and traditional approaches including: adjusting diet and fluid intake, local herbal remedies (namely *kastom lif* which literally means traditional leaf), prayer, massage, biomedicines (including paracetamol and antibiotics) and Oral Rehydration Therapy (ORT). Home treatments combine/involve both biomedical and traditional approaches, while traditional treatments entail prayer and kastom.

The rural healthcare worker witnessed participants bypassing the aid post for the hospital when seeking medical treatment for sick children. This is not surprising given rural health care workers have limited capacity to administer medicine (paracetamol and penicillin) and they cannot prescribe other antibiotics. The rural health care worker explained, “*I give them medication and I advise them as well. I advise them to keep their homes clean because coughs are sometimes caused by breathing in dust*… *Some people are allergic to Bactrim*, *therefore I advice them to squeeze lemon juice to their boiled water before they drink it*”.

It is common practice to combine biomedicine, home treatments and/or *kastom* medicine. Prayer, massage, paracetamol and *kastom* medicine in the form of *kastom lif* were used as the home treatment. The rural healthcare worker (P-16) urged people, “*to boil their drinking water*… *Moreover*, *I advised them to make sure that after playing*, *they must make sure that that their children wash their hands before they eat*…. *I think carelessness contributes to this as well*”. Yet biomedical treatment was almost always the preferred approach for perceived severe diarrheal illness in infants. *A mother* (*P-8) said “With diarrhea, some people know how to cure it with kastom medicine. However, with children as far as I am concerned, it is better to seek medical advice*”.

### Hand hygiene

Participants commonly described at least one of the biomedically-accepted indications for hand hygiene such as before eating, after toileting and before feeding a child. However, this knowledge did not routinely translate to normative behaviour. “*Laziness*” was the primary reason for low rates of domestic hand hygiene. Participants indicated that hand hygiene is not yet the social norm. A chief (P-21) said, *“People know they have to wash hands. However, people do not wash hands because they do not really care or they do not think it is important to wash their hands”.* The rural health worker (P-16) indicated her knowledge of hand hygiene was the result of attending workshops,*” Since I’ve been attending these workshops, I realize that you have to wash your hands any time after you’ve done or touched something that requires you to wash your hands. It is not only before you eat that you wash your hands. It is after sewing, after going to the toilet, before cooking or touching anything* (not clean) *that you should wash your hands”.*

## Discussion

We conducted an in-depth qualitative exploration of local understandings of general illness and diarrheal illness and its prevention for children for the first time in Vanuatu.

### Patterns of distress and explanatory models

In contrast to other studies [29] we found local explanations of general illness were enmeshed in and derived from both the traditional and biomedical system in no predetermined order. Participants classified illnesses according to a broad binary division of culture that is both biomedical and traditional (*kasto*m). When participants linked visible symptoms and imperceptible symptoms the illness was described as *kastom* illness. Yet causal explanations categorizing illness as biomedical or traditional often unfolded during the course and treatment of illness. People pragmatically seek to make sense of the illness experience drawing on whatever resources they have at hand at the time. They draw knowledge and understanding from both church and *kastom* systems that permeate every aspect of village life and thinking.

The features of some patterns of distress were well-known and lead to causation and therefore the treatment approach. This method of viewing illness is described by Beals [30] as “fixed-strategy” diseases (p. 1707). Diarrheal illness was commonly, but not always portrayed as a fixed-strategy illness. For success in improving health outcomes health education programs should incorporate local categorization with a biomedical understandings of diarrheal illness [31-34]. A local explanation of why illness occurs could be followed up with biomedical explanations of when and how illness could be treated.

### Patterns of help-seeking

Locals access traditional healers, home practitioners and biomedical practitioners differently depending on the patient and the symptoms. For small children, caregivers have a growing preference for biomedicine as the primary help-seeking option. Prior experience and evaluations of help seeking practice also influenced caregivers choice of health practitioner. Factors also determining illness help-seeking, included proximity of care, severity of illness, availability of medicines, and social and kin relationships.

For minor childhood diarrheal illness participants characteristically use home treatments. *Kastom* and local remedies are seen to be effective and accessible. Perceived diarrheal severity influenced out-of–home help-seeking. For severe childhood diarrhea help was sought earlier and more frequently from a biomedical option.

Practitioner choice appeared to be influenced by previous experience with severe illness. It was common practise for babies in Epau to have monthly health checks at the health dispensary. This suggests the community holds biomedicine in high regard. However, biomedicine has not entirely replaced other options. Another influence on the decision about which treatment route to pursue for childhood illness was the perceived efficiency of health practitioners [29]. Our rural healthcare worker enjoys great popularity as a healer; her approach is predominantly biomedical and is perceived to be effective. Kin relationships, and dominant local *kastom* or religious frameworks, boosted the rural healthcare workers popularity.

Engaging local healers serves to strengthen links between traditional medicine and biomedicine to ensure that local perspectives align with appropriate intervention aims and strategies [10,22,35]. Lambert reports [30] in other traditional cultures, together with biomedicine, many forms of local therapy continue to be widely used. In Epau people expressed a practical rather than ideological concern for help-seeking and using a combination of approaches was the norm. Exclusive reliance on either *kastom* or biomedical options was infrequent except for severe diarrheal illness. Our study highlights the acceptance of biomedical help seeking but it is not yet the default option for all severe childhood illness. Previous studies like ours have found communities are pragmatic and pluralistic, integrating new paradigms into their belief systems [22,29,30]. When a treatment is not perceived as being effective or lacked a desirable outcome then parents and caregivers seek alternative help.

### Specific treatment

Treatment choices are shaped by social roles and belief systems both individually and collectively. Treatments fall broadly into biomedical and traditional approaches consistent with other studies [29]. The cause of the illness is first determined so the right treatment can be selected. Where the causes of illness are spiritual a pastor or elder may use prayer. If the causes of illness are magic a *kleva* may be approached to treat the symptoms with *kastom lif*. Both practitioners and lay people may use treatments provided by the other domains, exposing the multiplicity of expertise and illustrating the difficulties drawing divisions across the health care system. If one treatment does not work then another treatment was commonly used. Syncretic use of treatments are also widely used as reported elsewhere in the Pacific [22,36]. Minor childhood diarrheal illness is characteristically treated with *kastom lif*, prayer, massage or paracetamol, while the biomedical practitioner treatment is ORT and/or antibiotics.

### Hand hygiene

Our findings that participants could describe biomedically-accepted indications for hand hygiene but that hand hygiene is not the social norm are not surprising. The same results are prevalent in high and low resourced settings world-wide [3,17,37,38]. Formative studies with mothers found nurturing, caring for and protecting babies to be a primary motivation for women to carry out hygiene activities [39]. The maternal response was to have clean hand when touching babies [40]. This suggests hand hygiene might best be promoted with Epau mothers during monthly child health checks at the rural dispensary.

## Conclusion

Vaughan [22] found patterns of distress in Tautua village on Malekula island, Vanuatu that were similar. Tautuan described illness as *stret sik* (biomedical) verses *ino stret sik* (traditional illness) [22]. In other parts of the world two medical systems have been found, a naturalistic model based in personal signs and systems and a faith model based on presumption aetiology [29].

Our study used purposive sampling, which included a small sample of consultants from Epau village. The findings may not be generalizable to other communities in Vanuatu. Our study population, intentionally skewed towards a caregiver and health practitioner perceptions, was not representative of the general village population. The interviewer had kinship ties with Epau village, which permitted interviews in Epau language but may have led to concerns about the confidentially of the survey responses. Our study reported local understandings of general illness and diarrheal illness including help-seeking, treatments and preventative behaviours. Self-reported behaviour may be over or under reported.

This study highlights some of the challenges in delivering childhood diarrheal illness care and promoting hand hygiene in Vanuatu. Belief about diarrheal illness was fluid and permeable influenced by both traditional and biomedical patterns of belief. The majority of respondents interviewed did not attribute diarrheal illness to bacterial infection, did not handwash and showed no concern for hand hygiene. Many people treated minor childhood diarrheal illness at home using traditional herbal remedies or traditional healers. Engaging traditional healers may strengthen the links between traditional and biomedicine to ensure that local perspectives on diarrheal illness signs, symptoms and treatments align with intervention aims and strategies. Inviting traditional healers to primary health training targeting diarrheal disease could activate hand hygiene messaging in the traditional healthcare arena, thus expose more people to messages. In support of formative studies showing mothers have a biological tendency toward nurture of babies, hand hygiene might best be promoted to Epau mothers during monthly child health visits at the medical dispensary. Promote hand hygiene at pertinent times such as disease outbreaks. Ni-Vanuatu mothers usually (but not always) recognize illness and make decisions about illness prevention, treatment and help seeking [21]. Hygiene has previously not been promoted at the aid post. The dispensary has neglected hygiene promotion in it’s add hoc approach to hygiene. Clear succinct hygiene messaging undertaken by the rural health care worker and biomedical practitioners at the dispensary would work to increasing hand hygiene rates and lower the incidence diarrheal disease in children in Epau community. Our study provides new evidence that there is a growing preference of biomedicine where biomedicine was the first preference for severe childhood diarrheal illness. We show how community based semi-structured surveying has potential be of use in community interventions to evaluate and monitor changes in conceptualisation of diarrheal disease and promotion of hand hygiene in community.

We found the local perspective challenges models that fail to consider local beliefs and practices. Biomedical professionals providing health service to Epau appear exemplars at this: biomedicine is the first preference for severely ill children. Why? Carers act pragmatically and perceive biomedicine as efficient, or they have a prior experience with child death. Diarrhea is known as fixed strategy disease that is normally treated biomedically when serious. Collaboration with local healers may help local healers introduce recommendations that the community will accept for childhood diarrheal illness. Hand hygiene messaging at the medical dispensary and by local healers would make appropriate use of existing community resources representing local traditions.

## Abbreviations

ORT: Oral rehydration therapy
P: Participant
WHO: World Health Organisation
